# Optimization of Mutation Pressure in Relation to Properties of Protein-Coding Sequences in Bacterial Genomes

**DOI:** 10.1371/journal.pone.0130411

**Published:** 2015-06-29

**Authors:** Paweł Błażej, Błażej Miasojedow, Małgorzata Grabińska, Paweł Mackiewicz

**Affiliations:** 1 Department of Genomics, Faculty of Biotechnology, University of Wrocław, Wrocław, Poland; 2 Section of Mathematical Statistics, The Faculty of Mathematics, Informatics and Mechanics, University of Warsaw, Warszawa, Poland; National Center for Biotechnology Information, UNITED STATES

## Abstract

Most mutations are deleterious and require energetically costly repairs. Therefore, it seems that any minimization of mutation rate is beneficial. On the other hand, mutations generate genetic diversity indispensable for evolution and adaptation of organisms to changing environmental conditions. Thus, it is expected that a spontaneous mutational pressure should be an optimal compromise between these two extremes. In order to study the optimization of the pressure, we compared mutational transition probability matrices from bacterial genomes with artificial matrices fulfilling the same general features as the real ones, e.g., the stationary distribution and the speed of convergence to the stationarity. The artificial matrices were optimized on real protein-coding sequences based on Evolutionary Strategies approach to minimize or maximize the probability of non-synonymous substitutions and costs of amino acid replacements depending on their physicochemical properties. The results show that the empirical matrices have a tendency to minimize the effects of mutations rather than maximize their costs on the amino acid level. They were also similar to the optimized artificial matrices in the nucleotide substitution pattern, especially the high transitions/transversions ratio. We observed no substantial differences between the effects of mutational matrices on protein-coding sequences in genomes under study in respect of differently replicated DNA strands, mutational cost types and properties of the referenced artificial matrices. The findings indicate that the empirical mutational matrices are rather adapted to minimize mutational costs in the studied organisms in comparison to other matrices with similar mathematical constraints.

## Introduction

Mutations occurring in DNA sequences are an inherent component of biological evolution. They, together with recombinations, generate a genetic variation which is subsequently subjected to selection. The most frequent mutations are substitutions, i.e., single nucleotide changes, which may be spontaneous, induced by radiation or chemicals, or introduced during the replication and repair of DNA. One of the most evident effects of mutations that arise during replication is DNA asymmetry. It manifests itself in different nucleotide and codon compositions of the diversely replicated DNA strands, called leading and lagging. This effect comes from various synthesis mechanisms of the DNA strands [1, 2], and is observed in most bacterial genomes, [3–11]. It has also significant consequences on various divergence rates of the genes located on the differently replicated DNA strands [12–17] as well as stability of their positions [18–20] and distribution on chromosomes [21, 22].

In practice, however, it is very hard to detect the effect of spontaneous mutations because many of them are eliminated by selection, especially those that happen in protein-coding sequences. Deleterious changes, such as nonsense mutations, which generate premature stop codons can lead to truncation of protein sequences. The harmfulness of other mutations, called missense, which change one coded amino acid to another depend on the differences in physicochemical properties of the substituted amino acids. The more these amino acids differ, e.g., in size, charge or hydrophobicity, the more harmful their replacement. Since many amino acids are encoded by two, three, four or six different codons, called synonymous, there are some mutations, called silent or synonymous, that do not change encoded amino acids and, consequently, protein composition and structure.

Mutations occurring in biological DNA sequences are the result of coevolution between mutational pressure and selection constraints around the genetic code [23–25], and can be optimized to some extent during evolution; see for review: [26, 27]. On the one hand, we should expect a tendency “for selection” to decrease the mutation rate because most mutations are deleterious and generate energetically costly repairing [28, 29]. On the other hand, mutations are responsible for genetic diversity, which is necessary for the adaptation of organisms to changing environments on the evolutionary scale. Therefore, an elevated level of mutation rate should be also expected in these cases [30–32]. This trade-off between the necessity to preserve accurate genetic information and requirements for adaptational flexibility indicates that some optimal mutation pressure can evolve [27, 33, 34]; however, this may depend on fitness landscape [35] and population structure [36].

The selection can operate to refine DNA replication and repair [37–43], which can influence the global mutation rate in organisms. An improvement of fidelity of DNA polymerases in DNA synthesis as well as effectiveness of their proofreading properties and post-replicative DNA mismatch repair mechanisms would decrease the general mutation rate. Otherwise, the rate would increase. The pattern of nucleotide substitution, i.e. relative rates of change from one type of nucleotide to another, can be also subjected to the optimization. For example, transitions, i.e., substitutions for the same chemical type of nucleotides, purine for purine or pyrimidine for pyrimidine, often cause fewer changes in coded amino acids or their properties than transversions, i.e., substitutions for different type of nucleotides, purine for pyrimidine and *vice versa*. Therefore, we can expect that a higher transitions/transversions ratio will be favored in mutational pressures.

The problem of optimization related to mutations has been studied in the context of the genetic code origin and its evolution [24, 25, 44–47]. It was postulated that many more assignments of codons to amino acids existed at the dawn of life on Earth; however, they were lost because they did not effectively minimize harmful effects of mutations on protein-coding sequences and translation errors. Optimization of codon usage has also been analyzed in terms of the reduction of deleterious mutational effects [46, 48, 49].

Since a significant fraction, usually more than 90% of bacterial genomes constitute protein-coding sequences [50], it is worthwhile to study the optimization of mutation pressure in respect to proteins. In this approach, we studied whether empirical mutational pressures expressed by transition probability matrices for particular bacterial genomes are better optimized to protein-coding sequences than other such types of matrices characterized by the same stationary distribution and the convergence speed to the stationarity. The optimization was considered according to probability of non-synonymous substitutions and different costs of amino acid substitutions occurring in products of protein-coding sequences.

## Materials and Methods

### Mutational transition probability matrices

We tested empirical mutational pressures described by transition probability matrices which were detected in six bacterial genomes by [15, 51, 52]. It is important in our studies to the matrices reflect neutral mutations in the absence of selection. To achieve that as much as possible, the authors in the inferring these matrices made a big effort to eliminate the potential influence of selection. The matrix for *Borrelia burgdorferi* was obtained by comparison of gene sequences with their potential pseudogenes found in intergenic regions [51], whereas matrices for *Escherichia coli*, *Chlamydia muridarum*, *Chlamydia trachomatis*, *Rickettsia*, *Staphylococcus aureus* and *Streptococcus pyogenes* genomes were inferred from comparison of synonymous sites in orthologous genes from closely related species or strains [15, 52]. However, there is a subset of highly expressed genes in which also synonymous substitutions are subjected to selection because of some preferences in codon usage, which is positively correlated with tRNA content in cells and the rate of translation [53–58]. Therefore, the authors removed the top 10% genes with most biased codon usage, expected to be the most highly expressed, to obtain the sites subjected to neutral substitutions. In our studies, we considered matrices for differently replicated DNA strand, leading and lagging separately, because they are subjected to various mutational patterns.

The matrices determine a unique homogeneous Markov chain, which characterizes the process of nucleotide substitutions and converges to the stationary distribution (Table 1). We used basic concepts of linear algebra and theory of Markov processes to investigate the properties of empirical matrices and define a class of artificial transition probability matrices *M* with similar properties. The artificial matrices were used as a reference to the empirical ones. The mathematical properties of the matrices are related to the stationary distribution and spectral decomposition of transition probability matrix [59–61].

**Table 1:** Nucleotide stationary distribution of leading and lagging strand matrices for studied genomes.

Genome	Leading strand				Lagging strand			
	A	T	G	C	A	T	G	C
*Borrelia burgdorferi*	0.32	0.49	0.14	0.06	0.49	0.32	0.06	0.14
*Chlamydia muridarum*	0.24	0.25	0.28	0.22	0.22	0.23	0.29	0.26
*Chlamydia trachomatis*	0.23	0.21	0.29	0.26	0.25	0.25	0.25	0.24
*Escherichia coli*	0.25	0.33	0.25	0.18	0.27	0.31	0.21	0.22
*Rickettsia* species	0.30	0.31	0.21	0.19	0.33	0.27	0.24	0.16
*Staphylococcus aureus*	0.41	0.39	0.12	0.08	0.35	0.45	0.09	0.11
*Streptococcus pyogenes*	0.33	0.42	0.12	0.13	0.30	0.40	0.09	0.20

It is well known from linear algebra and theory of Markov processes that every finite positive transition probability matrix *P* has a unique spectral decomposition

$$P=\text{A}\Lambda {\text{A}}^{-1},$$ (1)

where: A and A^-1^ are matrices whose rows/columns consist of right/left eigenvectors, respectively; Λ is a diagonal matrix with eigenvalues on its diagonal. The eigenvalues of the matrices are the solution of the characteristic equation. Therefore, there are four eigenvalues and four right/left eigenvectors for the matrix *P*. In general, some of the eigenvalues could be complex, not real numbers. The stationary distribution of the Markov process π is the left eigenvector (i.e., π*P* = π) and corresponds to the maximum of eigenvalues, which is always equal to 1 in the case of transition probability matrix. The second largest eigenvalue is responsible for the speed of convergence of Markov process to the stationary distribution, generated by *P*, which is a direct consequence of the Perron-Frobenius theorem [59–61]) Using these properties, we took into account the class *M* of transition probability matrices *P* = (*p*_*ij*_), *i*, *j* ∈ {*A*,*T*,*G*,*C*}, where *p*_*ij*_ denotes the probability of substitution from a nucleotide *i* to a nucleotide *j*, and *A*, *T*, *G*, *C* are nucleotides: adenine, thymine, guanine and cytosine, respectively. Each matrix *P* ∈ *M* is expressed by an equation:

$$P=\text{A}\Lambda {\text{A}}^{T}\Pi ,$$ (2)

where A is a real valued orthogonal matrix. Λ is a diagonal matrix with fixed the first and the second eigenvalue. Π is a diagonal matrix with the empirical stationary distribution π = {π_*A*_, π_*T*_, π_*G*_, π_*C*_} on its diagonal. A^-1^ = A^*T*^Π, which means that A is orthogonal in terms of stationary distribution. The Eq (2) is the special case of the Eq (1) and a general representation of the probability matrix *P* for a time-reversible Markov process [60]. Thanks to the Eq (2) it is very convenient to easily generate at random a sample of matrices from the class *M*, which is crucial from the computational point of view. Moreover, it is generally accepted in phylogenetic studies [62, 63] that the time reversible matrix is a very good description of the real substitution process and it is not necessary to apply more general unrestricted models with larger number of parameters, which could cause over-parameterization.

To search a wide class of possible alternatives to the empirical matrices in the class of time reversible Markov processes we applied the same stationary distribution and the same restrictions on some eigenvalues. We assumed that the second eigenvalues of artificial matrices are the same as in empirical matrices, which corresponds to the same time scale for stochastic processes generated by these matrices. In other words, it means that their stationary distributions converge with the same speed. In the representation (2), the third and the fourth eigenvalues are real variables. We tested three constraints on these eigenvalues to check a possible influence of these assumptions on obtained results: (i) all generated matrices had a constant probability of nucleotide substitutions under the stationary state (i.e., $\sum_{i\in \left\{A,T,G,C\right\}}\pi_{i}\left(1-p_{ii}\right)=const.$) as a corresponding empirical matrix (*constant* assumption), (ii) all generated matrices had the same eigenvalues as a corresponding empirical matrix (*equal* assumption), (iii) all generated matrices had the same sum of their eigenvalues as a corresponding empirical matrix (*trace* assumption).

### Fitness function

We considered several fitness functions *F* to investigate the influence of mutational matrices found for particular bacterial genomes on protein-coding sequences lying on the leading and lagging DNA strands in the corresponding genome (S1 Table). Sequences of these genes were downloaded from the GenBank database [64] and a decision about the location of these genes on the DNA strands was deduced according to the DNA asymmetry calculated in the Oriloc software [65]. Sequences from closely related genomes for which one mutational matrix was deduced were considered as one set.

In the functions *F*, we took into account the probability of non-synonymous substitutions and the mean value of amino acid substitution cost with and without nonsense mutations. Therefore, the optimizing matrices were tested on sites different from those used in inferring the empirical mutational matrices, which were derived from synonymous sites. The probability of non-synonymous substitutions was calculated based on the empirical codon frequency and the probability of change of one codon to another, coding different amino acids. The probability of the codon change was realized by a single nucleotide mutation based on the appropriate nucleotide substitution matrix. In the calculation of the mean value of the amino acid substitution costs, we additionally multiplied the probability of the codon change by a value reflecting differences between the amino acids. These differences were based on several amino acid scoring matrices and indices describing various physicochemical and biochemical properties of amino acids: chemical distance [66], hydropathy index [67], amino acid pair distance [68], EMPAR matrix [69] and polar requirement matrix [70]. All the matrices and indices were downloaded from the AAindex database [71].‎ The matrices and indices include only knowledge about properties of amino acids without any influence of underlying nucleotide mutations. When substitutions of stop codon were considered, we assumed their cost as the highest value of all amino acid substitution costs in the given measure.

To assess the optimality of empirical mutational matrix and easily compare the results for different genomes, we normalized the obtained values of fitness function according to the formula:

$$F_{norm}=\left(F_{emp}–F_{min}\right)/\left(F_{max}–F_{min}\right),$$ (3)

where: *F*_emp_ is the value of fitness function for the empirical matrix, *F*_min_ is the smallest and *F*_max_ the largest value of fitness function found for the artificial matrices under given conditions. Clearly, *F*_norm_ = 0 indicates that the empirical matrix minimizes costs of mutation just as the best artificial matrix, whereas *F*_norm_ = 1 means that the empirical matrix maximizes the effect of mutations just as the best generated matrix.

### Searching for the extreme values of fitness function

To find the maximum or minimum value of a given function *F* for artificial mutational matrices, we used the Evolutionary Strategies (ES) approach [72, 73]. This technique is an adequate tool in the finding solution of optimization problems, where the search space is not exactly defined and the solution is hard to find analytically. Similarly to the classical ES procedure, we started with the population of 100 random candidate solutions, i.e., transition probability matrices and carried out simulations according to the ES principles. The matrices at the initialization stage were computed according to the Eq (2). Three eigenvectors, which constitute matrix A, were selected at random and orthogonalized according to the Gramm-Schmidt orthogonalization procedure. The third and the fourth eigenvalue, which are necessary to well define the matrix Λ were selected at random based on one of three assumptions (see above). Unfortunately, this method does not guarantee to obtain always a probability transition matrix because negative elements can appear. Therefore, we had to repeat this procedure up to the valid probability matrix was generated.

At each generation step, we applied a mutation and selection procedure. The process of mutation introduction was realized by a modification of a matrix (an individual), which was done by a random shift of eigenvectors and/or two eigenvalues according to the normal distribution *N*(0,σ). The scale parameter σ = 0.3 was tuned in initial simulation tests to obtain a quick convergence to the optimal solution. We run the Gramm-Schmidt orthogonalization procedure so many times to obtain a probability transition matrix.

In the selection stage, the half of the worst candidate solutions (according to their *F* values) were deleted and replaced by survived individuals. Simulations were run over 10,000 steps. The length of the simulations proved sufficient because all important parameters stabilized till this time (S1 Fig). Finally, the best matrices under a given criterion were extracted from the population.

In S1 Fig, we presented a typical simulation run, which was described by the average, minimum and maximum value of the fitness function *F*, calculated from the population of 100 individuals in every generation. It is clear that all considered statistics increased sharply about the 500th simulation steps and then remained stable until the end of the simulation despite small fluctuations of the minimum. To check the stability of the algorithm, we compared simulations with the same parameters but under different random seeds (S2 Fig). These simulations converged to the same value of fitness function.

Comparisons of empirical and optimized matrices by Principal Component Analysis (PCA) and Kruskal-Wallis test were carried out in Statistica (StatSoft Inc. 2011, version 10, www.statsoft.com). In PCA, we assumed a covariance matrix in the calculation of principal components.

## Results

The aim of our study was to test to what extent spontaneous mutational pressures described by transition probability matrices are optimized according to harmful effects on protein-coding sequences. We considered six mutational matrices inferred from various bacterial genomes (S1 Table). Their effects were measured by probability of non-synonymous substitutions and costs of amino acid substitutions with and without nonsense mutations. The amino acid costs were described by different matrices and indices, which characterized various physicochemical and biochemical properties of amino acids. As a reference set to the empirical mutational matrices, we used optimized matrices that were initially randomly generated. For their generation, we applied spectral representation of time reversible Markov processes, which fulfilled the assumed conditions. Our model allowed us to control several parameters of the Markov process such as the speed of convergence to the stationary distribution and probability of substitutions in the stationary state. The generated matrices had the same stationary distribution as well as the first and the second eigenvalues as the empirical matrices. However, the third and the fourth eigenvalue were chosen according to three additional claims. The constant assumption meant that all generated matrices had the constant probability of nucleotide substitutions under stationarity as a corresponding empirical matrix. The equal assumption indicated that all generated matrices had all the same eigenvalues as the empirical matrix. Finally, the trace assumption meant that all generated matrices had the sum of their eigenvalues as the empirical matrix. Using the modified Evolutionary Strategy approach, we searched the space of randomly generated matrices to find the best optimized matrices, i.e., maximizing or minimizing harmful effects on protein-coding sequences expressed by a fitness function. Finally, the empirical mutational matrices were compared with the solutions found.

### General comparison of empirical and optimized matrices

**Fig 1:**
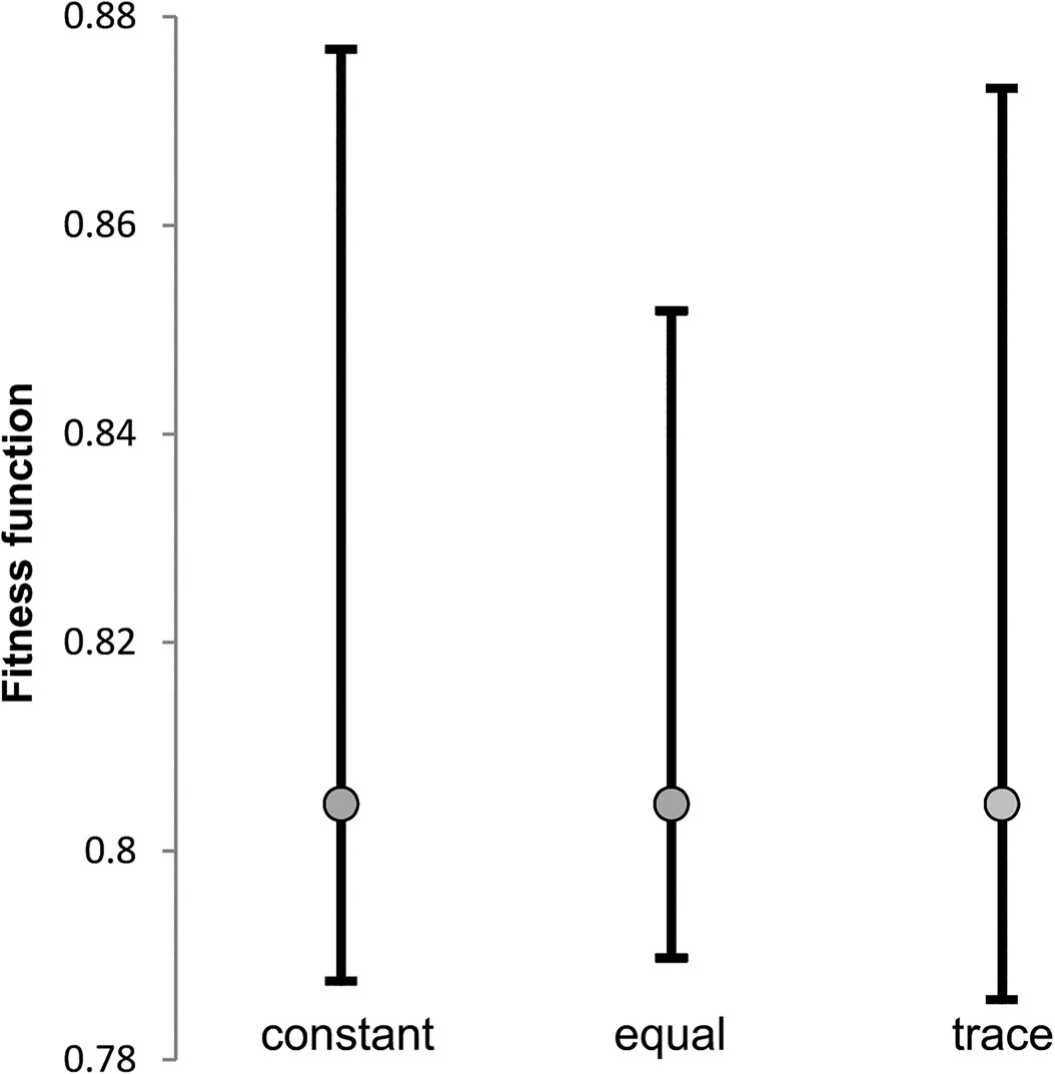
Fitness function for three assumptions on eigenvalues. Comparison of the fitness function (measured by the probability of non-synonymous substitutions) for the *B*. *burgdorferi* mutational matrix from the leading strand (grey dot) with the largest (upper whiskers) and the smallest (lower whiskers) values found for artificial matrices considering three types of assumptions on eigenvalues.

In the example shown in Fig 1, we presented values of a given fitness function *F* for three types of assumptions on eigenvalues. It is visible that the value for empirical leading strand matrix from *Borrelia burgdorferi* is located closer to the smallest fitness function than to the largest value. It indicates that the empirical matrix is to some extent optimized to minimize the probability of non-synonymous substitution. To easily compare results between different genomes, measures of mutation effect and assumptions for the third and fourth eigenvalues, we normalized the value of fitness function comparing empirical matrix value with values for optimized matrices (Eq 3). Briefly, the normalized fitness function (*F*_norm_) approaching 0 indicates that the empirical matrix has a tendency to minimize costs of mutations, whereas *F*_norm_ approaching 1 means that this matrix maximizes the effect of mutation on protein-coding sequences. Sample results for selected conditions were shown in Tables 2 and 3.

**Table 2:** The normalized fitness function for non-synonymous substitutions.

Genome	Assumption on eigenvalues		
	constant	equal	trace
*Borrelia burgdorferi*	0.190	0.238	0.215
*Chlamydia muridarum*	0.170	-0.004	0.161
*Chlamydia trachomatis*	0.161	0.042	0.155
*Escherichia coli*	0.128	-0.010	0.124
*Rickettsia* species	0.249	0.131	0.254
*Staphylococcus aureus*	0.270	0.206	0.287
*Streptococcus pyogenes*	0.238	0.163	0.284

The normalized fitness function was measured by the probability of non-synonymous substitutions for leading strand matrices from seven genomes and three types of assumptions on eigenvalues.

**Table 3:** The normalized fitness function for costs of amino acid substitutions.

Genome	Assumption on eigenvalues					
	constant		equal		trace	
	AA	AA+Stp	AA	AA+Stp	AA	AA+Stp
*Borrelia burgdorferi*	0.266	0.209	0.015	0.010	0.144	0.121
*Chlamydia muridarum*	0.182	0.193	0.246	0.265	0.221	0.221
*Chlamydia trachomatis*	0.216	0.231	0.320	0.360	0.217	0.234
*Escherichia coli*	0.125	0.165	0.074	0.147	0.058	0.145
*Rickettsia* species	0.225	0.218	0.176	0.171	0.198	0.201
*Staphylococcus aureus*	0.245	0.279	0.019	0.017	0.142	0.109
*Streptococcus pyogenes*	0.289	0.210	-0.001	0.048	0.115	0.114

The normalized fitness function measured by the mean cost of amino acid substitutions without (AA) and with (AA+Stp) stop codons using polar requirement for the leading strand matrices from seven genomes and three types of assumptions on eigenvalues.

**Fig 2:**
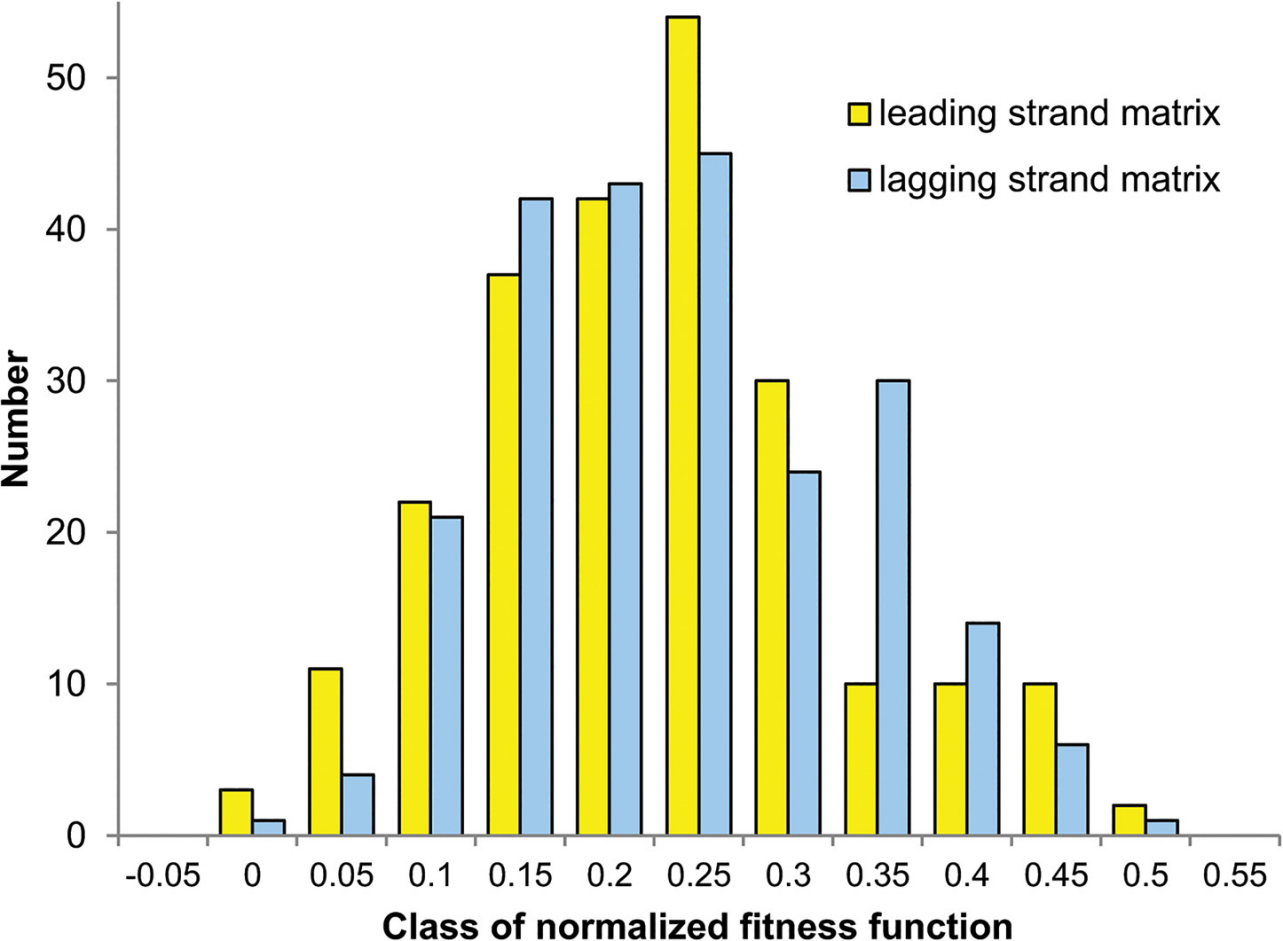
Distribution of normalized fitness function for empirical matrices. The distribution of normalized fitness function *F*_norm_ for all 231 combinations of genomes, assuming different measures of mutational effect on protein-coding genes and three assumptions on eigenvalues of generated matrices for two differently replicated DNA strands.

Fig 2 presents a distribution of *F*_norm_ values for all 231 combinations of genomes, different measures of mutational effect on protein-coding genes and three assumptions on eigenvalues of the generated matrices for two DNA strand separately. For all these combinations, the empirical mutational matrices were closer (*F*_norm_ was lower than 0.5) to the artificial matrices minimizing costs of mutations than to the matrices maximizing them. The leading strand matrices were slightly better optimized (the mean *F*_norm_: 0.202, the range: -0.010 to 0.487) than the lagging strand matrices (the mean *F*_norm_: 0.212, the range: -0.006 to 0.471). The empirical leading strand matrices showed *F*_norm_ < 0.25 in more than 73% of tested conditions, whereas the lagging strand matrices in more than 67% of conditions. It indicates slightly better minimization of the leading strand matrices. The largest *F*_norm_ value (0.487) was for the *Chlamydia trachomatis* leading strand matrix compared with the best artificial matrices optimized according to the equal assumption on eigenvalues and the costs of amino acids substitutions considering their hydrophobic properties. What is more, in four cases, the applied algorithm found no better artificial matrix than the empirical one, which performed slightly better than the best artificial matrix (*F*_norm_ obtained negative values from -0.001 to -0.01). Such empirical matrices were the leading strand matrix for *Streptococccus pyogenes* tested according to the costs of amino acids substitutions under polar requirement, as well as the lagging strand matrix for *C*. *trachomatis* and the leading strand matrices for *C*. *muridarum* and *Escherichia coli* tested according to the probability of non-synonymous substitution. All these instances fulfilled the equal assumption on eigenvalues, i.e., the most restrictive one, which can explain the close similarity between the empirical and found optimized matrices.

**Fig 3:**
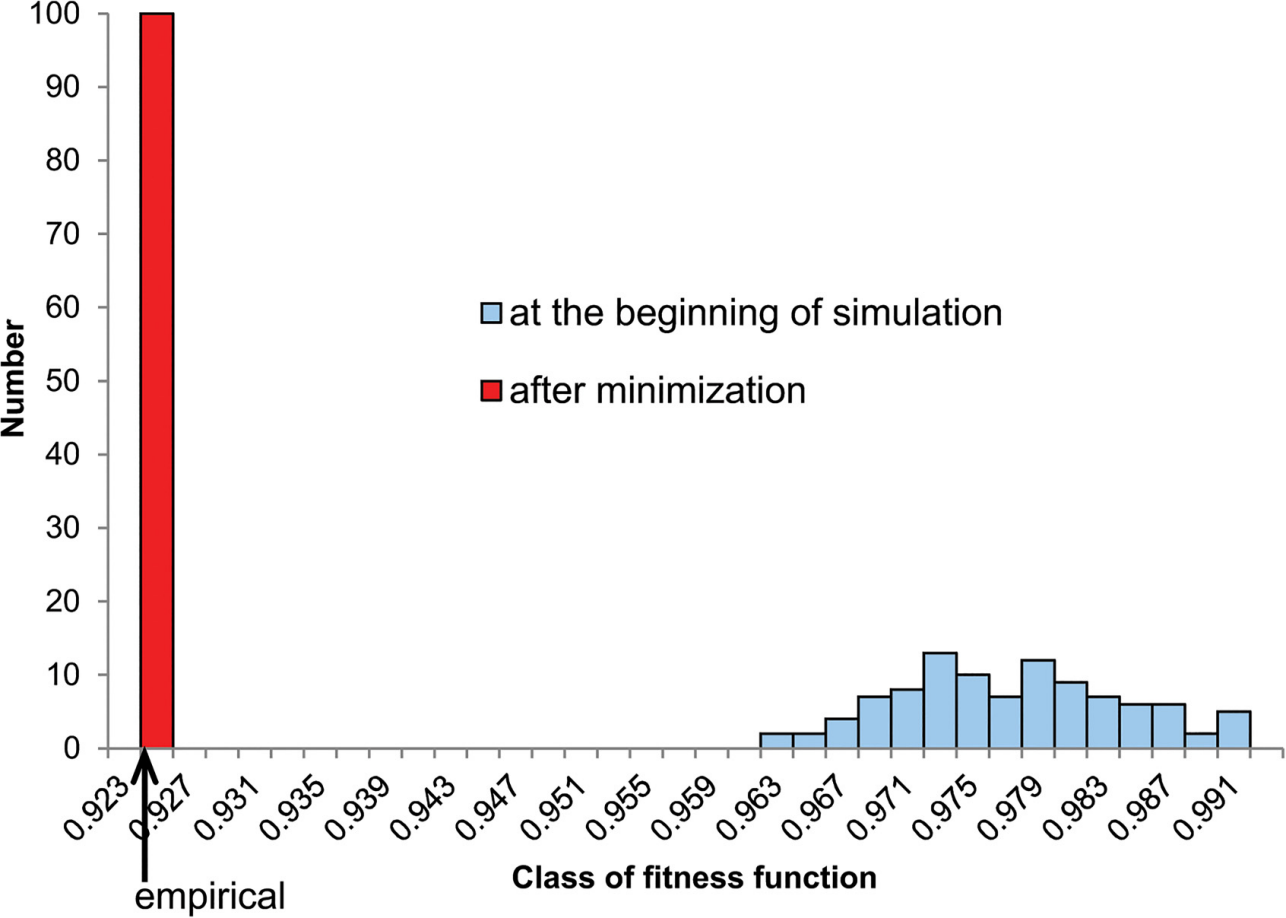
Distribution of fitness function for artificial matrices for *S*. *pyogenes*. The distribution of fitness function *F* for 100 artificial starting matrices (with equal assumption) at the beginning of simulation and after minimization (1000 steps) according to the costs of amino acids substitutions under polar requirement. The value for the empirical leading strand substitution matrix from *S*. *pyogenes* (0.923161) was indicated by the arrow.

**Fig 4:**
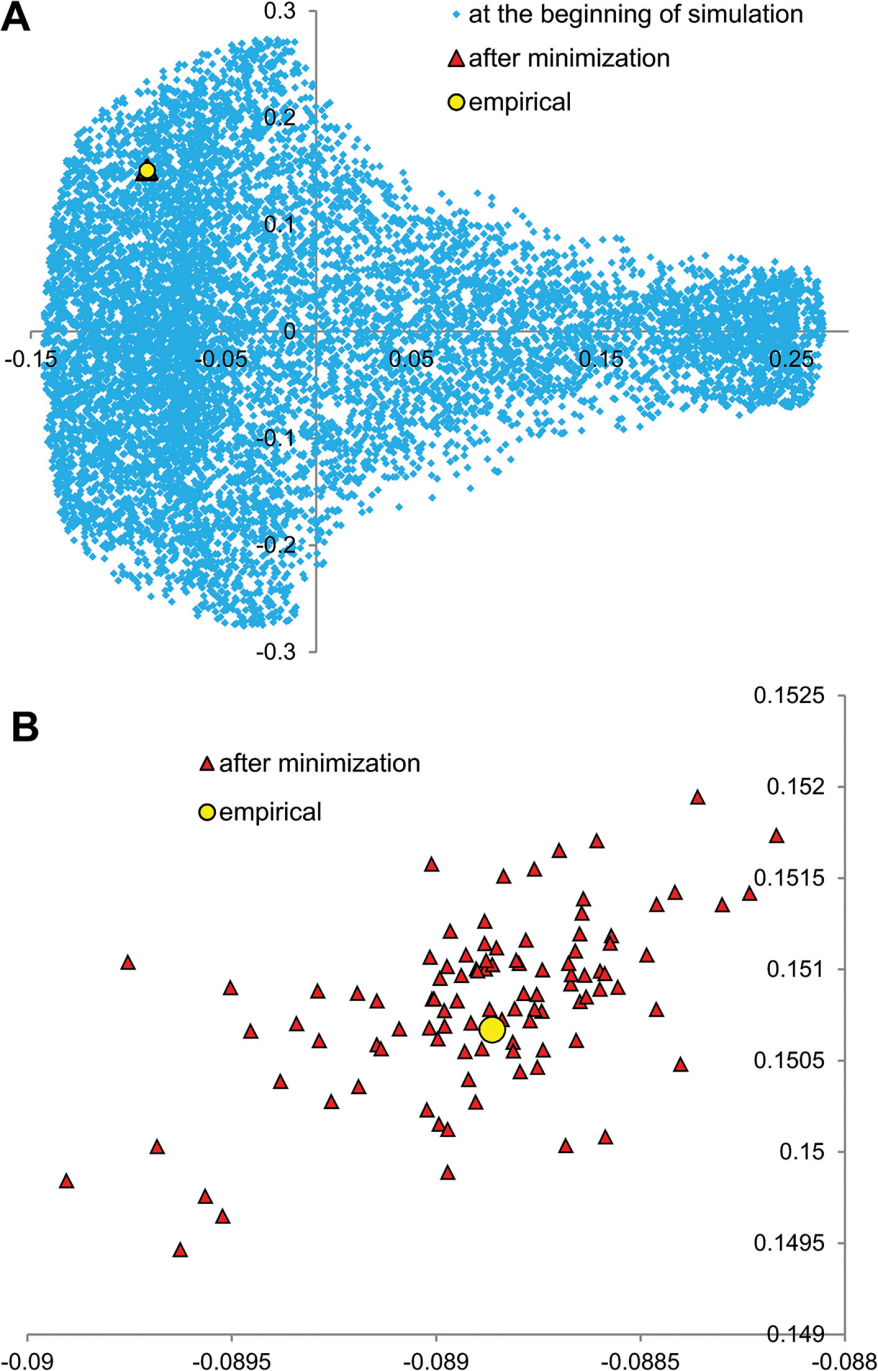
PCA of *S*. *pyogenes* empirical matrix and artificial matrices. (A) The Principal Component Analysis of artificial starting matrices (with equal assumption) at the beginning of simulation and after the minimization according to the costs of amino acids substitutions under polar requirement. The empirical leading strand substitution matrix from *S*. *pyogenes* was indicated by the open circle. (B) The enlarged part of A focused on the region occupied by the minimizing and empirical matrices.

To check if in these four cases the algorithm got stuck in a local minimum and was unable to find better solution, we carried out 100 additional simulations with different seeds. However, none of them produced matrices that were better optimized than the empirical ones. Moreover, the algorithm always converged almost to the same solution. The percentage difference between extreme values of fitness function of these solutions was extremely small, from 0.002% (for *S*. *pyogenes*) to 0.026% (for *E*. *coli*). We also checked if initial matrices were, in fact, randomly generated and represented a wide range of starting points for the algorithm to search a vast space of potential solutions. In fact, the range of their fitness function was from 46 (for *E*. *coli*) to 1326 (for *S*. *pyogenes*) times larger than the found solutions (Fig 3). In Fig 4, we also visualized the matrices by Principal Component Analysis (PCA), which showed that the initial matrices represented a wide spectrum of starting points, whereas the minimizing matrices were restricted to a very small region. Interestingly, the empirical matrix was placed in the middle of the solutions found.

We observed a small difference in the value of normalized fitness function *F*_norm_ between genomes. Considering three types of assumptions on eigenvalues, *E*. *coli* matrices from two DNA strands were on average better optimized according to the probability of non-synonymous substitutions (0.06) than *Chlamydia* matrices (0.11) and other genomes (0.21 – 0.28). However, considering costs of amino acids substitutions, *Chlamydia* matrices were on average slightly worse (0.27 and 0.28 for *C*. *muridarum* and *C*. *trachomatis*, respectively) than other genomes (0.17 – 0.19). Nevertheless, these results indicate that there are no significant differences between optimization of matrices coming from different genomes and tested on different fitness functions.

A small difference in *F*_norm_ was found considering assumptions on the third and fourth eigenvalues of the generated matrices. For the probability of non-synonymous substitutions and the equal assumption, *F*_norm_ was nearly on average two times smaller (0.13) than for constant (0.20) and trace assumptions (0.23) for all considered matrices. For different costs of amino acid substitutions, the normalized fitness function was on average larger for the constant assumption (0.26) than for the trace and the equal assumptions (0.18 for these two cases). Small values for the equal assumption can result from the most restrictive conditions on eigenvalues.

Considering different measures of mutation effects on protein-coding sequences, we found that the empirical matrices were generally the best optimized according to EMPAR matrix [69] (*F*_norm_ = 0.16), next to polar requirement [70] (0.17) and non-synonymous substitutions (0.18). A slightly higher value of *F*_norm_ = 0.24 was obtained for chemical distance [66], hydropathy index [67] and amino acid pair distance by [68]. We did not find a significant difference between *F*_norm_ calculated for amino acid costs with or without nonsense mutations, with the exception of the equal assumption (0.20 vs 0.17, respectively).

### Properties of empirical and optimized matrices

**Fig 5:**
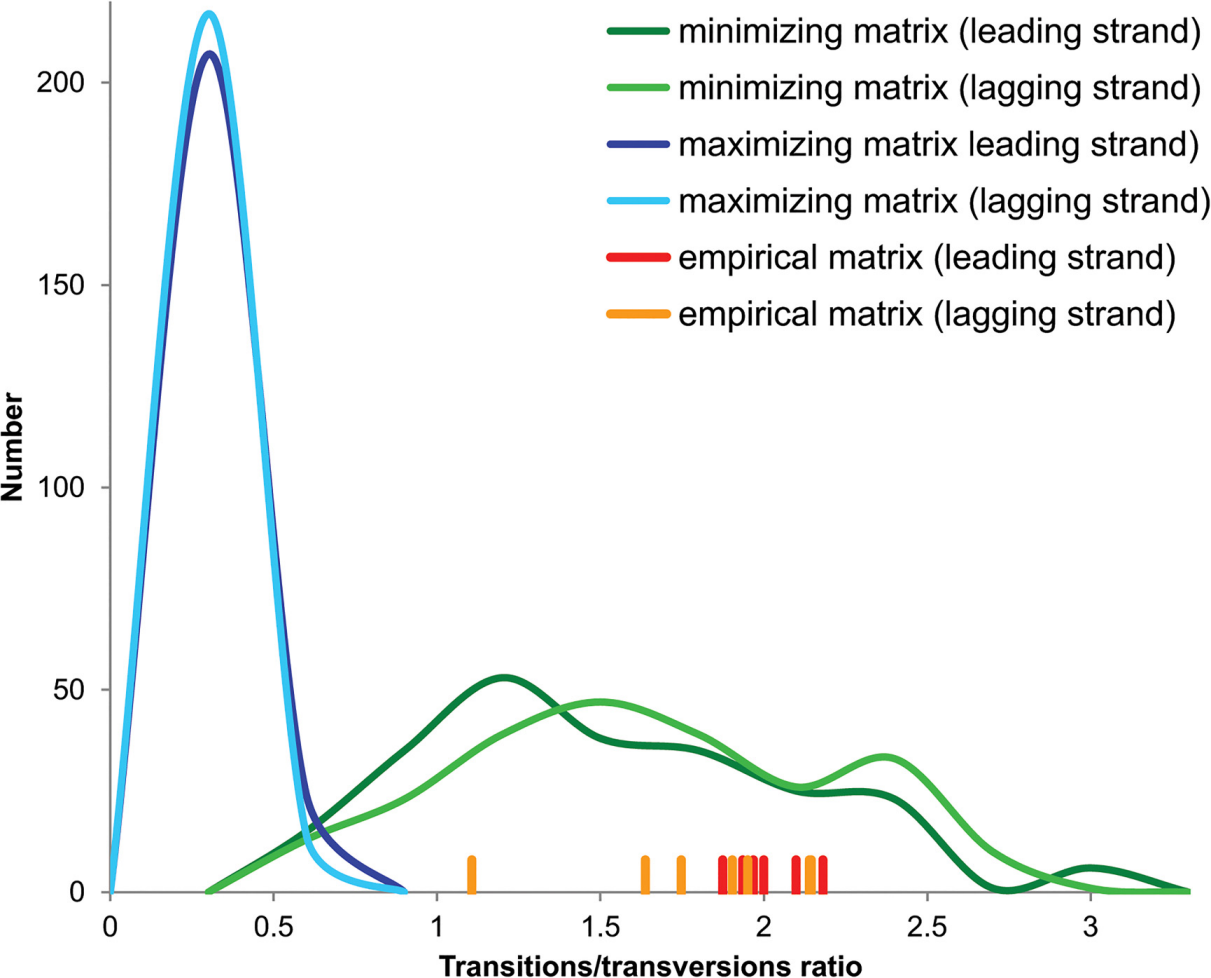
Transitions/transversions distribution. The distribution of transitions to transversions ratio for empirical matrices as well as matrices maximizing and minimizing costs of amino acid and non-synonymous substitutions. Data for two differently replicated DNA strands (leading and lagging) were considered, separately.

One of parameters that was used to describe spontaneous mutational pressure is transitions to transversions ratio. The expected ratio should be 1:2, if all nucleotide substitutions happen with the same probability. However, transitions are usually observed several times more often in real sequences than transversions [74]. The observed bias results from higher rate of chemical changes between structurally similar nucleotides and more probable transition substitutions introduced during DNA replication. In addition to that, transitions are less harmful than transversions in terms of changing coded amino acids or their properties and, therefore, more often accepted. Thus, we should observe that matrices minimizing costs of amino acid and non-synonymous substitutions are characterized by the high transitions/transversions ratio compared to maximizing matrices. Actually, the ratio for the minimizing matrices was on average about nine to ten times larger than for maximizing ones and did not differ significantly between matrices for two DNA strands (Table 4). Interestingly, the average ratio for empirical matrices was even bigger than that for the minimizing matrices. Although the distribution of the ratio for the minimizing matrices was quite wide, almost 72% of the values for the leading strand matrix were larger than one and, likewise, 80% of the values for the lagging strand matrix were also greater than one (Fig 5). In contrast to that, none of the maximizing matrices exceeded this value and all but one were smaller than 0.5. The empirical matrices well corresponded to the distribution of the minimizing matrices.

**Table 4:** The ratio of transitions to transversions for different types of matrices.

	Empirical matrices		Minimizing matrices		Maximizing matrices	
	leading	lagging	leading	lagging	leading	lagging
Mean	1.88	1.80	1.38	1.49	0.15	0.14
Minimum	1.11	1.11	0.39	0.38	0.00	0.00
Maximum	2.18	2.14	2.99	2.75	0.53	0.46

The transitions/transversions ratio was calculated for seven empirical matrices and, in the case of optimizing matrices, for all 231 combinations of genomes, assuming different measures of mutational effect on protein-coding genes and three assumptions on eigenvalues.

**Fig 6:**
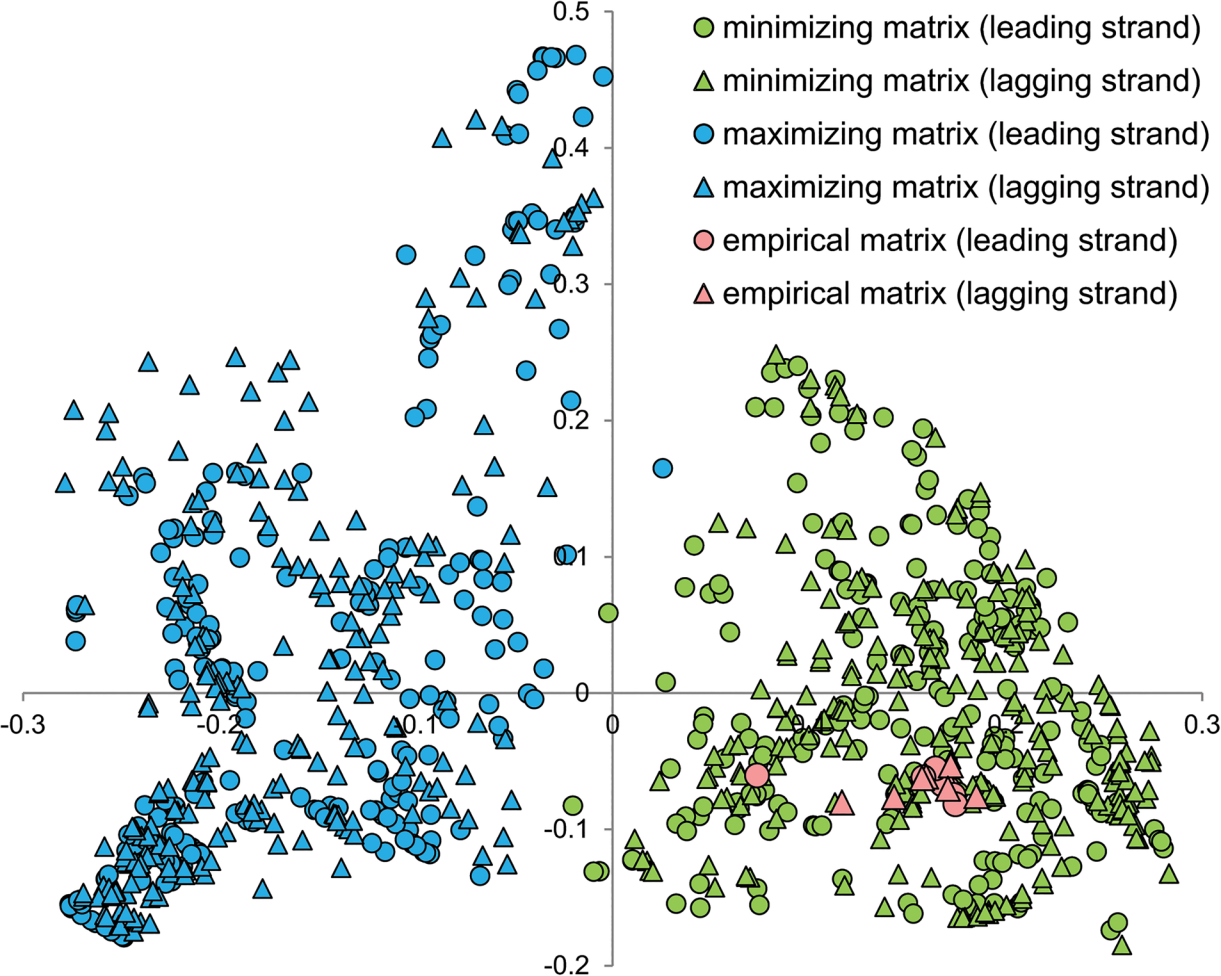
PCA of empirical and optimized artificial matrices. The Principal Component Analysis of the empirical substitution matrices as well as 231 matrices maximizing and minimizing costs of amino acids and non-synonymous substitutions. Data for two differently replicated DNA strands (leading and lagging) were considered, separately.

Examples of optimized matrices in comparison to corresponding empirical matrix were presented in S2 Table. To easily visualize matrices according to all possible 12 nucleotide substitutions (corresponding to elements of these matrices), we carried out Principal Component Analysis to reduce the number of dimensions from 12 matrix elements to two main variables (Fig 6). Maximizing and minimizing matrices were clearly separated into two non-overlapping groups by the first component, which indicates that they differ in their elements. All empirical matrices were placed among the set of minimizing matrices, which indicates a similar pattern (rate) of nucleotide substitutions described by the empirical and the optimized matrices. There was also no difference in the distribution of matrices in respect to differently replicated DNA strands. The largest correlation with the first (most discriminative) component showed transversions: A→T (-0.73), T→A (-0.72), G→T (-0.69) and T→G (-0.59), as well as transitions A→G (0.75) and G→A (0.80). The opposite signs at the correlation coefficients are connected with a different influence of these variables on the first component and the separation of sets.

**Fig 7:**
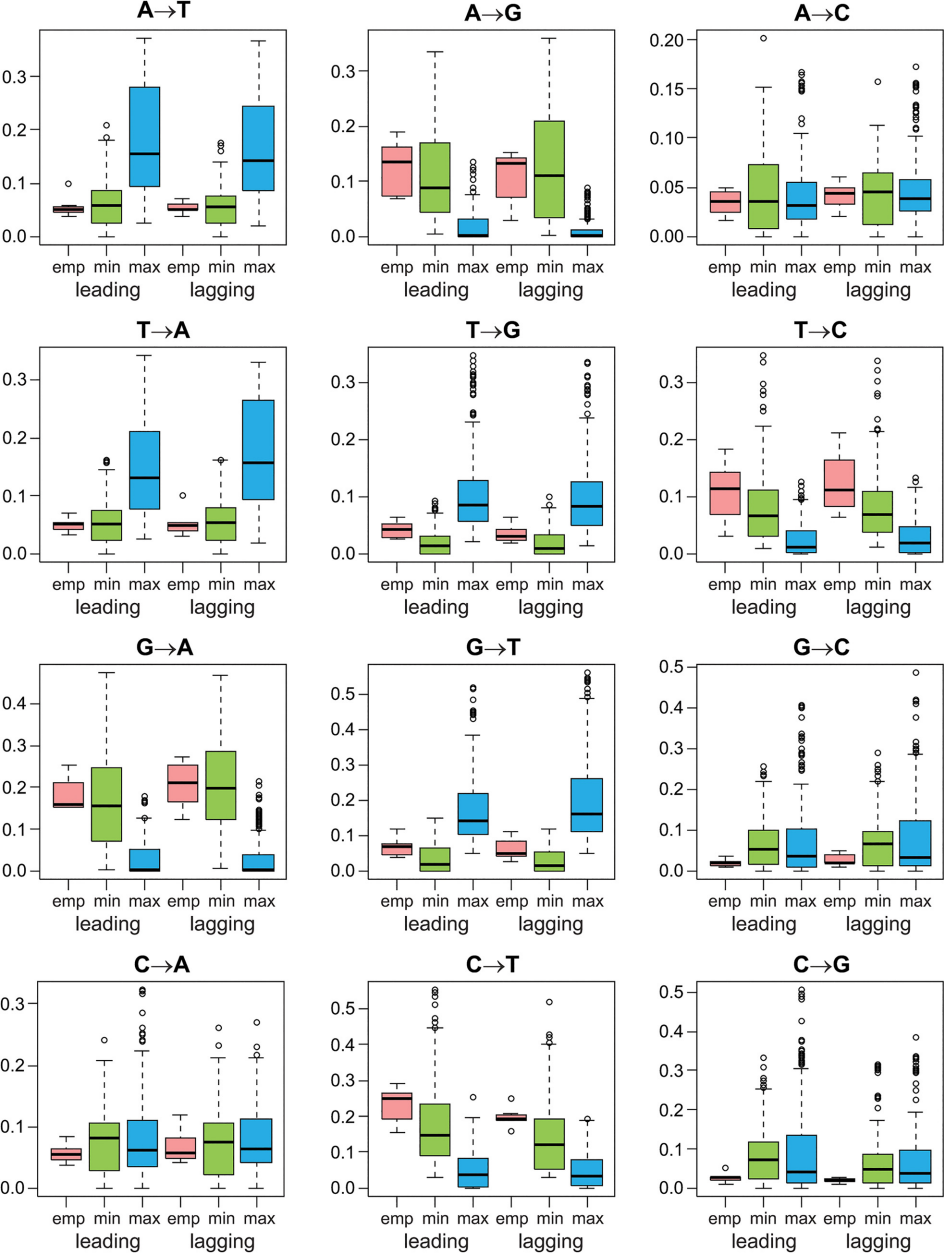
Box-plots of nucleotide substitutions’ rates for empirical and optimized artificial matrices. The thick horizontal lines indicate median, the colored boxes show quartile range and the whiskers determine the range without outliers. Data for two differently replicated DNA strands (leading and lagging) were considered, separately.

The transversions A→T and T→A had statistically significantly (Kruskal-Wallis test, p-value < 0.001) smaller rates in the analyzed empirical and minimizing matrices than in the maximizing matrices, whereas the transitions A→G, G→A, T→C and C→T were significantly larger in the first-mentioned matrices than in the latter (p-value < 0.0032) (Fig 7). It is worth emphasizing that we did not find significant differences between the empirical and minimizing matrices for any of these substitutions. Transitions T→G and G→T also showed smaller values for the empirical and minimizing matrices than the maximizing ones. Differences between the minimizing and the maximizing matrices for these two substitutions and between the empirical and maximizing matrix for the lagging strand in the case of G→T were statistically significant (p-value = 0.043). The other substitutions did not show significant differences between the matrices in almost all cases, although the empirical matrices were characterized by the smallest rate of transversions G→C and C→G.

Three most frequent substitutions of the minimizing leading and lagging strand matrices were the same but differed in proportions. These were transitions: C→T, G→A and A→G, with percentages 34%, 33% and 18% for the leading strand and 26%, 52% and 11% for the lagging strand (S3 Table). The same substitutions dominated also in four, one and three of seven empirical matrices from the leading strand, respectively. For the lagging strand matrices, C→T and G→A dominated in two and four cases, respectively. In contrast to that, these three substitutions were selected as the rarest substitution in 53% (for the leading strand) and 61% (for the lagging strand) of maximizing matrices. In turn, three the least frequent substitutions were T→G (32%), A→C (20%) and G→C (14%) in the minimizing leading strand matrices, whereas in the minimizing lagging strand matrices, T→G (39%), C→G (17%) and A→T (11%). The T→G and A→C transversions had also the smallest rate in two empirical leading strand matrices and G→C substitution in five of them. In the case of the empirical lagging strand matrices, four had C→G and three G→C as the rarest substitution but none T→G or A→T. However, three the least frequent substitutions in 66% of the minimizing leading strand matrices were selected as the most dominant mutation in only 13% of maximizing matrices (A→C in none of them). The proportions for the lagging strand matrices are 57% and 37%, respectively. The lack of the full correspondence between the rarest substitutions in the minimizing matrices and the most common substitutions in the maximizing ones, as could be expected, may result from the same restrictions imposed on the generated matrices, e.g., stationary distribution and the same speed of convergence to the distributions.

## Discussion

The minimization of harmful effects of mutations can be achieved by a decrease in the global mutation rate by evolution of high-fidelity polymerases, which select and incorporate nucleotides into newly synthesized DNA strands [38, 39, 75]. The other adaptations can be more effective mechanisms of mutation correction: exonucleolytic proofreading [43] and post-replicative DNA mismatch repair [40, 41], which excise and replace incorrectly inserted bases. Here we focused on more sophisticated aspects of this optimization, namely on the pattern of nucleotide substitutions, i.e., relative rates between changes of particular nucleotides. Such optimization can be connected with changes in the quantity of nucleotides in the cellular dNTP pools [76, 77] as well as preferences of polymerases and correction mechanism to particular nucleotides [78–84], which may favor introduction of one nucleotide over another to DNA.

The nucleotide substitution patterns are usually described by transition probability matrices. To assess the optimization of the empirical mutational matrices, we compared them with the reference set of optimized artificial matrices. In contrast to the method used by Błażej et al. [23], in which the class of General Time Reversible matrices with six parameters was considered as the reference set, here we analyzed a more convenient class of transition probability matrices. In both cases, the generated matrices had the stationary distribution (the left eigenvector corresponding to the first eigenvalue) as the empirical matrix. However, in this approach we also assumed the second eigenvalue (corresponding to the speed of convergence to the stationarity) as in the empirical matrices, while the third and fourth eigenvalues could vary. Thus, the empirical mutational matrices were compared with other matrices in their class with similar mathematical properties.

The obtained results indicate that spontaneous mutational pressures in bacterial genomes, described by transition probability matrices, are optimized to minimize rather than maximize the frequency of non-synonymous substitutions and costs of amino acid replacements according to their different physicochemical and biochemical properties. In all 231 analyzed cases, the influence of mutational pressure on protein-coding sequences in comparison to the best optimized artificial matrices, measured by the normalized fitness function, never exceeded 0.5 (in this scale, 0 indicates that the empirical matrix minimizes costs of mutations as the best artificial matrix, whereas 1 means that this matrix maximizes costs of amino acid substitutions as the best artificial matrix). Interestingly, in four cases the empirical matrix minimized the influence of mutation on protein-coding sequences slightly better than any optimized matrices. We thoroughly tested if the applied algorithm got stuck in a local minimum during searches of the best solution. However, 100 simulations with different seeds always converged to the same or very similar solution.

It is interesting that empirical nucleotide mutation matrices showed a similar trend in the minimization of harmful substitutions, despite generating different stationary distributions and testing on different protein-coding sequences. This trend did not depend, or depended very weakly, on the analyzed genomes, effects of mutations on protein-coding sequences and assumptions on eigenvalues of artificial optimized matrices, to which the empirical ones were compared. It indicates that various mutational pressures are similarly optimized to minimize costs of mutations in different biological systems. We also did not find significant differences between matrices from two differently replicated DNA strands (leading and lagging), although the leading strand matrices were slightly better minimized than matrices from the lagging strand. It may be related with the preferred location of genes that are essential for cell functioning (e.g., coding for ribosomal proteins) in the leading strand [21, 22]. The genes for ribosomal proteins maintain conserved positions on bacterial chromosomes with the phylogenetic distance of compared genomes [18]. Moreover, it was observed a higher frequency of gene translocations from the lagging to the leading strand rather than in the opposite direction [19] and smaller rate of substitutions’ accumulation in the leading than lagging strand genes [12–15].

Both the empirical matrices and matrices minimizing mutational effects on protein-coding sequences demonstrated the excess of transitions over transversions as it would be expected because the latter have more harmful impact on proteins. Although the leading and lagging strand matrices for the same genome are characterized by different nucleotide stationary distributions (Table 1), they also showed similar patterns of nucleotide substitutions with the large rate of transitions A→G, G→A, T→C and C→T as the minimizing matrices. It indicates that the minimization of mutational costs is realized by the same relative rates.

Although most mutations are deleterious (especially those replacing amino acids with different properties), we should not expect the perfect minimization of mutational effects by empirical matrices. The mutational pressure can approach two extremes, the minimization and maximization [26, 27]. The pressure is minimized to decrease the number of harmful mutations and cost of DNA repair. On the other hand, the pressure can be maximized to increase a genetic variation and the number of profitable substitutions driven by positive selection, which are necessary in changing environmental conditions and strong competition between organisms. The mutations balance between these two extremes and obtain some optimal values specific for a given biological system (genome) [23]. In agreement with that, the analyzed empirical mutational matrices, when compared with the optimized matrices, locate between these two extremes showing, however, closer similarity to matrices that minimize costs of mutations.

## Supporting Information

S1 Fig Variation of fitness function. The minimum, maximum and mean of fitness function *F* for the rate matrix maximizing amino acid costs and characterizing by a constant probability of nucleotide substitutions as the empirical leading strand matrix from *B*. *burgdorferi* (the constant assumption).
(PDF)

S2 Fig Fitness function for 10 runs. The mean value of fitness function *F* for ten runs of the algorithm with different seeds aimed to find the rate matrix maximizing amino acid cost and characterizing by a constant probability of nucleotide substitutions as the empirical leading strand matrix from *B*. *burgdorferi* (the constant assumption).
(PDF)

S1 Table Genomes and their protein-coding genes used in the study. (DOCX)

S2 Table *S*. *pyogenes* empirical matrix and optimized artificial matrices. The comparison of the empirical matrix from *S*. *pyogenes* leading strand with matrices optimized according to the costs of amino acids substitutions under polar requirement and equal assumption on eigenvalues. The substitutions were sorted in descending order according to the empirical values.
(DOCX)

S3 Table Number of matrices in which a given substitution showed the largest or the smallest rate for the leading (before slash) and lagging (after slash) DNA strands. (DOCX)
