# Supplementary figures and images for: Optimization of Mutation Pressure in Relation to Properties of Protein-Coding Sequences in Bacterial Genomes
Source: PLoS One. 2015 Jun 29;10(6):e0130411. doi: 10.1371/journal.pone.0130411 (PMC4488281; doi:10.1371/journal.pone.0130411)

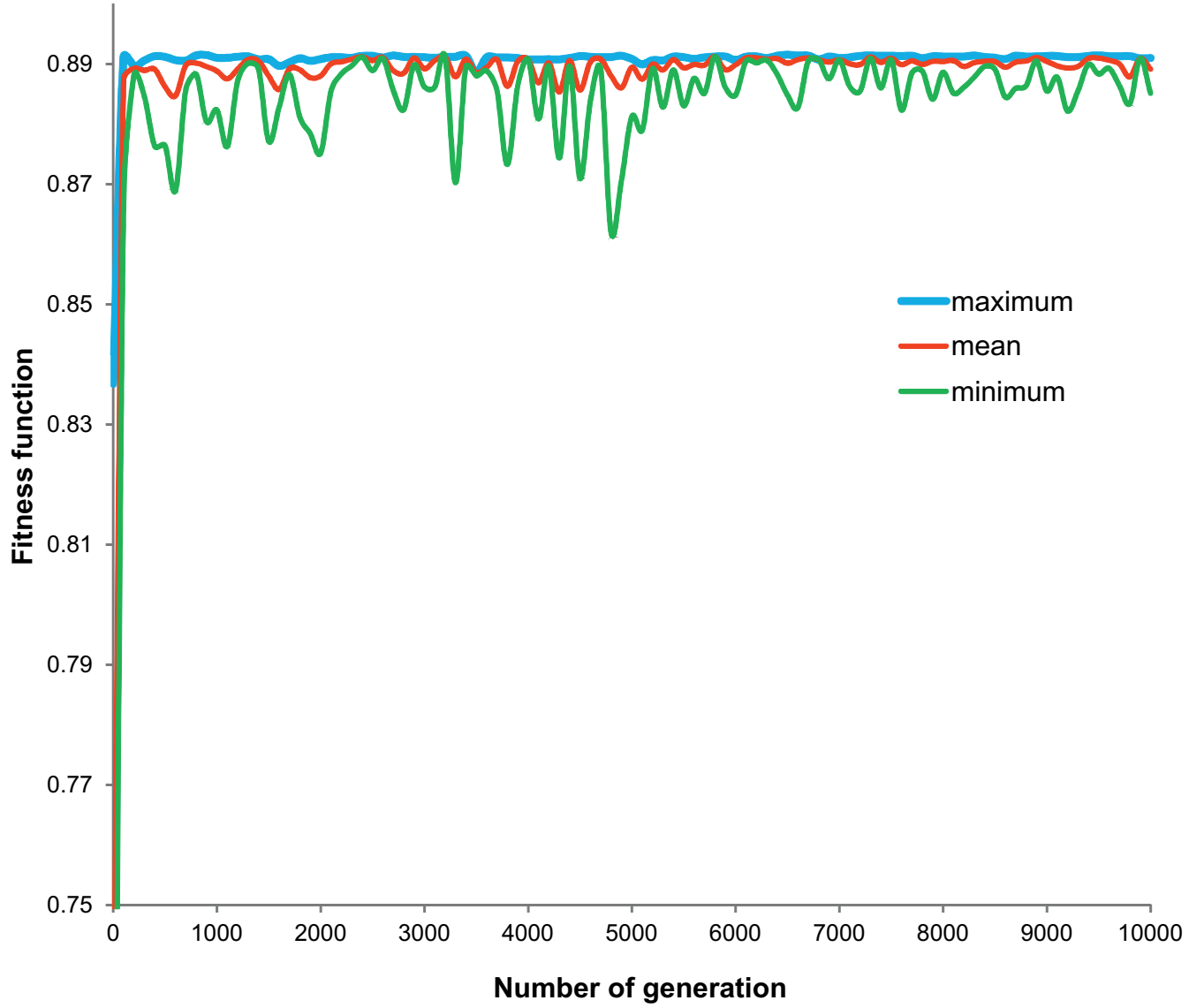

Supplement: S1 Fig — The minimum, maximum and mean of fitness function F for the rate matrix maximizing amino acid costs and characterizing by a constant probability of nucleotide substitutions as the empirical leading strand matrix from B. burgdorferi (the constant assumption). (PDF) [file pone.0130411.s001.pdf]

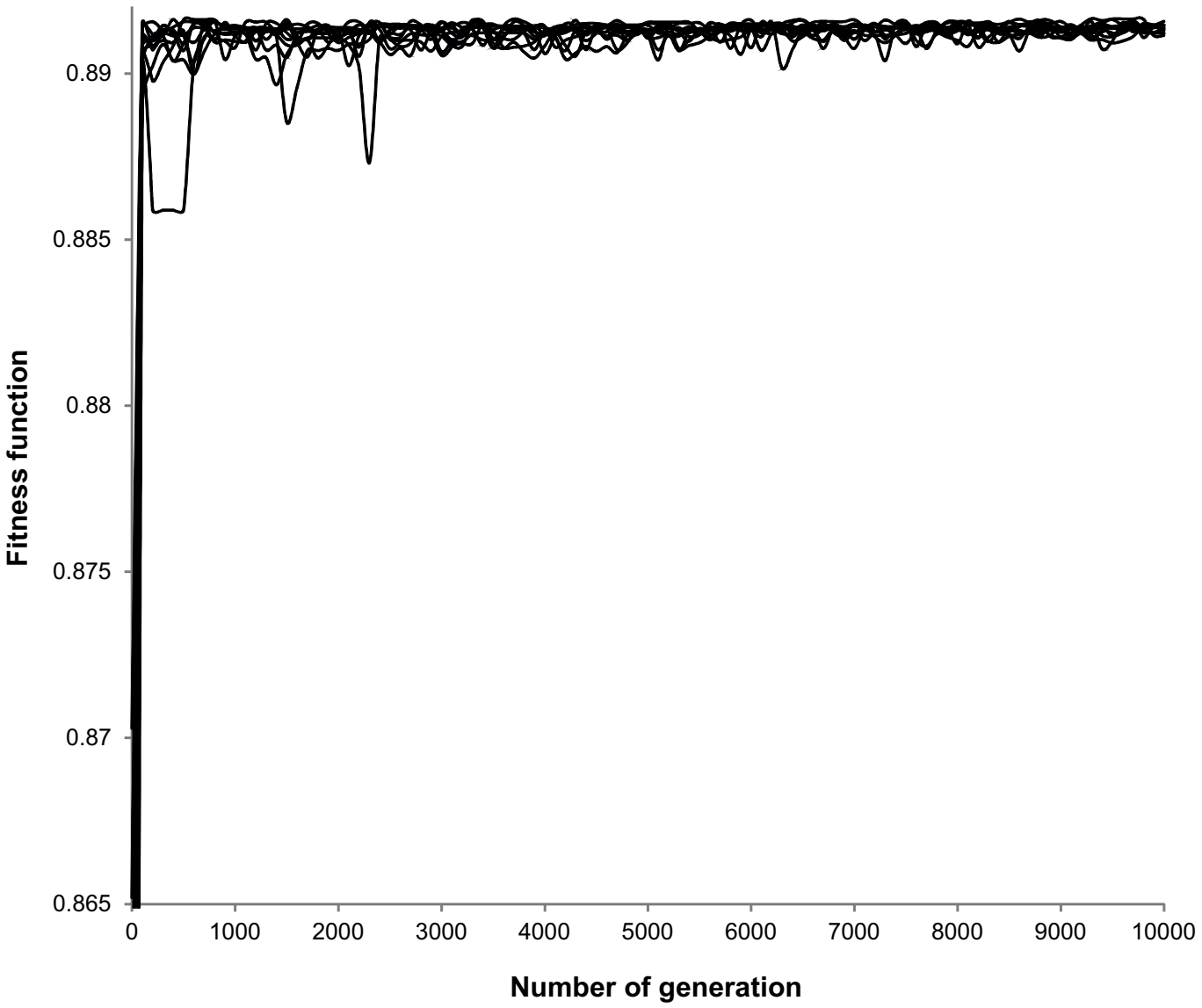

Supplement: S2 Fig — The mean value of fitness function F for ten runs of the algorithm with different seeds aimed to find the rate matrix maximizing amino acid cost and characterizing by a constant probability of nucleotide substitutions as the empirical leading strand matrix from B. burgdorferi (the constant assumption). (PDF) [file pone.0130411.s002.pdf]
