# Supplementary material for: Optimization of Mutation Pressure in Relation to Properties of Protein-Coding Sequences in Bacterial Genomes
Source: PLoS One. 2015 Jun 29;10(6):e0130411. doi: 10.1371/journal.pone.0130411 (PMC4488281; doi:10.1371/journal.pone.0130411)
Supplement: S1 Table — (DOCX) [file pone.0130411.s003.docx]

**S1 Table. Genomes and their protein-coding genes used in the study.**

| **Genome** | **Accession number** | **Number of genes on strand** | | **Total gene length on strand [bp]** | |
| --- | --- | --- | --- | --- | --- |
|  |  | **leading** | **lagging** | **leading** | **lagging** |
| *Borrelia burgdorferi* B31 | NC_001318 | 333 | 141 | 356,034 | 173,796 |
| *Chlamydia muridarum* Nigg | NC_002620 | 512 | 407 | 539,778 | 436,323 |
| *Chlamydia trachomatis* D/UW-3/CX | NC_000117 | 507 | 395 | 513,497 | 434,802 |
| *Escherichia coli* str. K-12 substr. MG1655, O157:H7 str. Sakai, CFT073, 042 | NC_000913, NC_002695, NC_004431, NC_017626 | 11198 | 8577 | 10,375,123 | 7,799,045 |
| *Rickettsia prowazekii* Madrid E, *R. typhi* Wilmington, *R. felis* URRWXCal2, *R. conorii* Malish 7 | NC_000963, NC_006142, NC_007109, NC_003103 | 2688 | 1764 | 2,364,246 | 1,588,938 |
| *Staphylococcus aureus* N315, MRSA252, COL | NC_002745, NC_002952, NC_002951 | 5908 | 1933 | 5,497,047 | 1,547,097 |
| *Streptococcus pyogenes* SF370, SSI-1, MGAS8232, MGAS6180, MGAS315, MGAS10394 | NC_002737, NC_004606, NC_003485, NC_007296, NC_004070, NC_006086 | 8834 | 2193 | 7,895,787 | 1,803,942 |
