# Supplementary material for: Optimization of Mutation Pressure in Relation to Properties of Protein-Coding Sequences in Bacterial Genomes
Source: PLoS One. 2015 Jun 29;10(6):e0130411. doi: 10.1371/journal.pone.0130411 (PMC4488281; doi:10.1371/journal.pone.0130411)
Supplement: S2 Table — The comparison of the empirical matrix from S. pyogenes leading strand with matrices optimized according to the costs of amino acids substitutions under polar requirement and equal assumption on eigenvalues. The substitutions were sorted in descending order according to the empirical values. (DOCX) [file pone.0130411.s004.docx]

**S2 Table. *S. pyogenes* empirical matrix and optimized artificial matrices.**

| **Substitution** | **Empirical** | **Minimizing** | **Maximizing** |
| --- | --- | --- | --- |
| C→T | 0.291 | 0.271 | 0.000 |
| G→A | 0.208 | 0.182 | 0.116 |
| T→C | 0.084 | 0.084 | 0.000 |
| G→T | 0.082 | 0.107 | 0.213 |
| A→G | 0.076 | 0.068 | 0.044 |
| C→A | 0.056 | 0.060 | 0.142 |
| A→T | 0.045 | 0.043 | 0.198 |
| T→A | 0.039 | 0.033 | 0.154 |
| G→C | 0.035 | 0.049 | 0.008 |
| A→C | 0.029 | 0.024 | 0.057 |
| C→G | 0.028 | 0.046 | 0.007 |
| T→G | 0.027 | 0.031 | 0.062 |

The comparison of the empirical matrix from *S. pyogenes* leading strand with matrices optimized according to the costs of amino acids substitutions under polar requirement and equal assumption on eigenvalues. The substitutions were sorted in descending order according to the empirical values.
