# Supplementary material for: Optimization of Mutation Pressure in Relation to Properties of Protein-Coding Sequences in Bacterial Genomes
Source: PLoS One. 2015 Jun 29;10(6):e0130411. doi: 10.1371/journal.pone.0130411 (PMC4488281; doi:10.1371/journal.pone.0130411)
Supplement: S3 Table — (DOCX) [file pone.0130411.s005.docx]

**S3 Table. Number of matrices in which a given substitution showed the largest or the smallest rate for the leading (before slash) and lagging (after slash) DNA strands.**

| **Substitution** | **the largest rate** | | | **the smallest rate** | | |
| --- | --- | --- | --- | --- | --- | --- |
|  | **empirical matrices** | **minimazing matrices** | **maximizing matrices** | **empirical matrices** | **minimazing matrices** | **maximizing matrices** |
| A→C |  |  |  | 1/0 | 46/24 | 7/1 |
| A→G | 2/0 | 42/26 |  |  |  | 77/108 |
| A→T |  | 5/4 | 63/56 |  | 16/25 |  |
| C→A |  | 1/3 | 7/7 |  | 2/8 | 5/0 |
| C→G |  | 19/9 | 36/6 | 0/4 | 4/40 | 6/20 |
| C→T | 4/2 | 79/59 |  |  |  | 6/5 |
| G→A | 1/4 | 76/119 |  |  |  | 39/28 |
| G→C |  | 1/5 | 5/13 | 5/3 | 32/8 | 28/7 |
| G→T |  |  | 69/72 |  | 24/16 |  |
| T→A |  | 2/0 | 27/53 |  | 29/21 |  |
| T→C | 0/1 | 6/6 |  |  | 4/0 | 63/61 |
| T→G |  |  | 24/24 | 1/0 | 74/89 | 0/1 |
